# Effects of repeated sprint training on repeated sprint ability and short-distance sprint performance in youth soccer players: a meta-analysis

**DOI:** 10.1186/s13102-026-01869-5

**Published:** 2026-07-15

**Authors:** Ziyi Lin, Qun Yang, Xiang Gao, Xuyan Cui

**Affiliations:** 1 School of Sport Training, Guangzhou Sport University, Guangzhou, China; 2 Faculty of Health Sciences and Sports, Macao Polytechnic University, Macao, China; 3 Guangdong Provincial Key Laboratory of Human Sports Performance Science, Guangzhou Sport University, Guangzhou, China

**Keywords:** Repeated sprint training, Soccer players, Anaerobic sprint ability, Meta-analysis

## Abstract

Sprinting ability is crucial in soccer, determining a player’s performance in key scenarios such as repeated high-speed changes of direction, pressing, and tracking back. This systematic review and meta-analysis aimed to evaluate the effects of repeated sprint training (RST) on sprinting performance in youth soccer players. A systematic search was conducted across Chinese and English databases, including CNKI, PubMed, Web of Science, and Google Scholar, resulting in the inclusion of seven studies. The meta-analysis revealed that, compared to regular training, RST produced a significant improvement in RSA_mean_ (*SMD* = -0.79, 95% *CI*: -1.40 to -0.18, *P* = 0.010) and short-distance sprint performance (*SMD* = -0.59, 95% *CI*: -0.92 to -0.26, *p* < 0.001). In contrast, the effect of RST on RSA_best_ (*SMD* = -0.08, 95% *CI*: -0.40 to 0.24, *p* = 0.60) was similar to that of regular training, with no significant difference. In conclusion, RST effectively enhances mean repeated sprint and short-distance sprint performance in youth soccer players. When implementing RST, factors such as the athlete’s age, fitness level, and training period should be taken into account.

**Supplementary Information:**

The online version contains supplementary material available at 10.1186/s13102-026-01869-5.

## Introduction

Soccer has evolved into an increasingly fast-paced sport, imposing greater physical demands on players and necessitating a superior capacity for high-intensity exercise [1–5]. During match play, athletes must frequently execute anaerobic actions: such as acceleration, deceleration, braking, and repeated sprints, which are often decisive for the outcome and require the ability to reproduce maximal power output within short time frames [6–8]. According to the principle of training specificity, optimal physical preparation should incorporate short-duration, high-intensity, and sport-specific drills that closely mimic the demands of competition. In this context, high-intensity training methods like sprint interval training have been shown to improve soccer players’ sprint performance and lower-limb power [9–11]. However, these methods are truly not optimal for developing Repeated Sprint Ability (RSA), as they fail to provide the specific movement and metabolic stimuli required for this multifaceted quality [12, 13].

Repeated Sprint Training (RST) is characterized by short-duration (< 10 s), maximal-intensity sprints interspersed with brief recovery periods (< 60 s) [14, 15]. By enhancing anaerobic capacity, power, and lactate tolerance, RST has emerged as a superior training modality in soccer conditioning. It demonstrates superior efficacy over continuous endurance training in improving both RSA and short-distance sprint performance [16, 17].

Recent comprehensive systematic reviews and meta-analyses have established the overall effectiveness of RST for improving physical fitness in athletes [18–20]. Specifically, Thurlow et al. [18–20] synthesized evidence across multiple team sports, confirming that RST enhances repeated sprint ability. Furthermore, the effectiveness of RST is highly dependent on its programming variables. Typical RST protocols involve sprint distances of 20–40 m, 4–10 repetitions per set, 2–5 sets, with inter-sprint rest periods of 15–30 s and inter-set rest of 1–10 min. Weekly training frequency ranges from 1 to 3 sessions, and total intervention duration from 4 to 10 weeks [18–20]. However, the analysis by Thurlow et al. [18–20] included heterogeneous athletic populations and did not focus exclusively on soccer players. Moreover, the differential effects of RST on RSA_best,_ RSA_mean_ and on short-distance sprint performance, have not been quantitatively synthesized within the specific context of youth soccer. Consequently, the dose–response relationship of these programming variables—particularly their interactive effects on RSA_best_ and RSA_mean_—remains poorly understood in youth soccer populations. Additionally, the influence of key training prescription variables (e.g., intervention duration, sprint distance, rest intervals) on these outcomes remains underexplored in this population.

To address these gaps, this study conducted a comprehensive literature search and employed a meta-analysis. The primary objectives were: (1) to determine the effects of RST, compared with conventional training, on RSA_best_, RSA_mean_ and short-distance sprint performance; (2) to quantify the influence of key programming variables on these effects through subgroup analyses; (3) to clarify the practical value of RST for enhancing anaerobic sprint capacity in youth soccer players. We hypothesized that RST would lead to significant improvements in RSA_mean_ and short-distance sprint performance—which are more dependent on the ability to resist fatigue and repeated power output—while its effect on RSA_best_, a measure of single maximal effort, might be limited compared to conventional training.

## Method

### Literature search and selection criteria

This systematic review and meta-analysis aimed to evaluate the effects of Repeated Sprint Training (RST) on sprint performance in soccer players, based on the current body of literature. The study was conducted in accordance with the PRISMA 2020 guidelines and was prospectively registered on PROSPERO (registration number: CRD420251238363). Inclusion and exclusion criteria were established using the PICO framework [21], with the full details presented in Table 1.

**Table 1:** Inclusion criteria (PICO framework)

population	Intervention	Comparison	Outcome
Types of Athletes: Soccer players; Youth soccer players Level of Play: Elite; Semi-professional; Amateur	Training Type Repeated Sprint Training: RST Training plan Intervention duration ≥ 4 weeks, training frequency ≥ 1 session/week, intensity ≥ 90% of maximum sprint speed	Regular soccer training (e.g., technical drills, tactical practice, small-sided games) without structured RST.	Key indicators Repeated Sprint Ability: RSA_best_; RSA_mean_ Short-distance sprinting ability: 10 m,20 m,30 m

Outcome Measures:①RSAbest：The time (s) recorded for the fastest single sprint within a set of repeated sprints (typically 6—10 × 20—40 m, with 15—30 s of rest between sprints)；②RSAmean：The average time (s) of all sprints completed within the same set of repeated sprints；③Short-distance Sprint Performance：The time (s) for a single, all-out linear sprint over distances of 10 m, 20 m, or 30 m.For all the above metrics, a lower value indicates better performance

Exclusion Criteria: 1)Duplicate publications; 2)Non-peer-reviewed or unpublished material e.g., conference abstracts, advertisements; 3)Studies for which the full text was unavailable; 4)Studies with an unclear or unspecified intervention protocol; 5)Studies lacking relevant outcome data.

### Search strategy

**Fig. 1:**
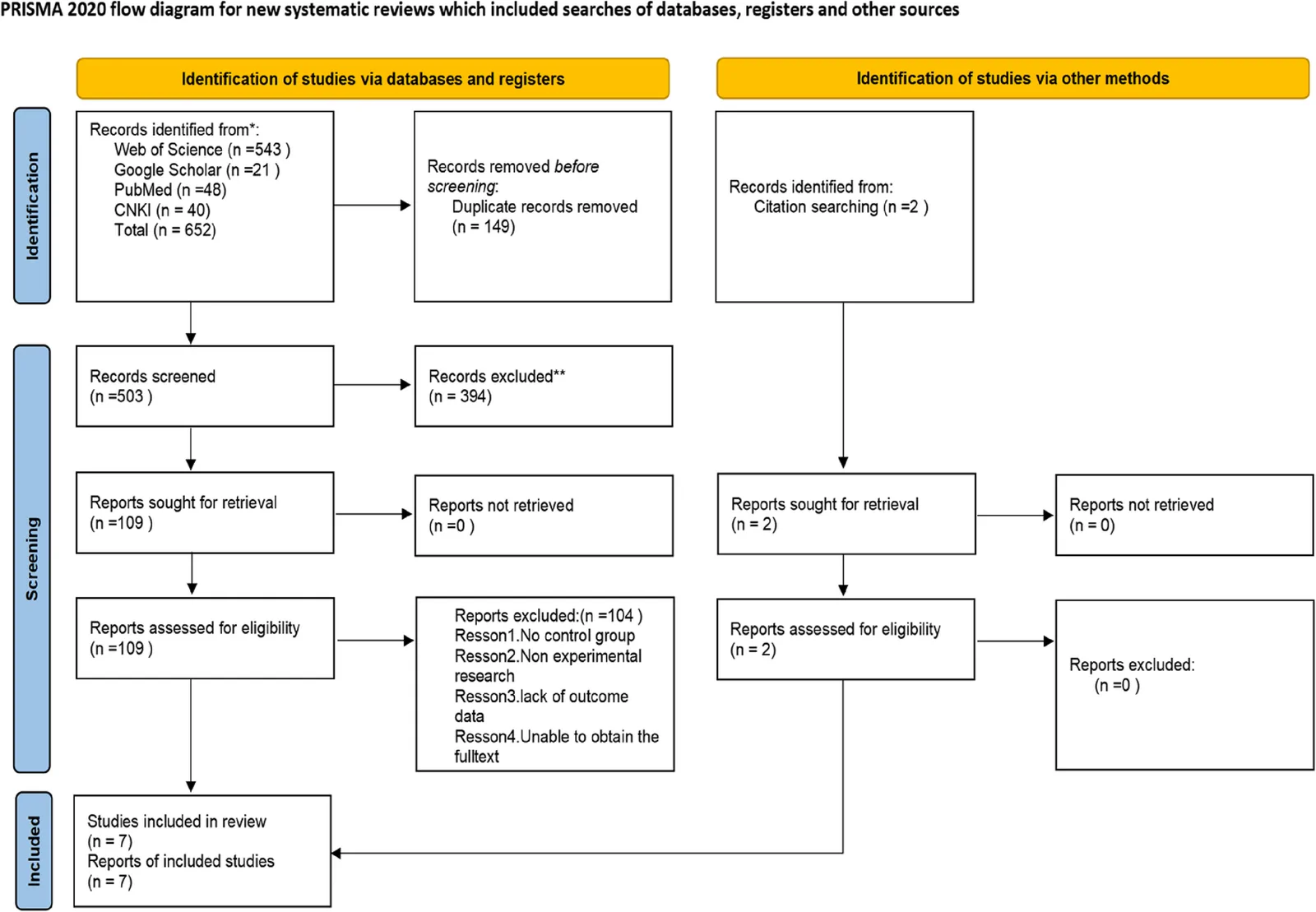
Literature screening flowchart

The Chinese database utilized CNKI as its retrieval platform, with search terms including “repeated sprint training”, “repetitive sprint training”, “sprint interval training”, “speed endurance training”, and “repeated sprint capacity”. The search of international databases, including PubMed, Web of Science, and Google Scholar, utilized the following keywords: “repeated sprint training”, “repeated-sprint training”, “sprint interval training”, “speed endurance training”, “anaerobic endurance training”, “repeated sprint ability”, and “sprint ability”. These terms were used individually or in combination. The initial search, last updated on December 20, 2025, identified 652 records. The literature screening and selection process was conducted independently by two reviewers. This process was conducted independently by two reviewers. Initially, both reviewers screened the titles and abstracts of all identified records against the predefined inclusion and exclusion criteria. Potentially eligible studies were retrieved for full-text assessment. Subsequently, the two reviewers independently evaluated the full-text articles to determine final inclusion. Any discrepancies or disagreements at any stage of the screening process were resolved through discussion between the two reviewers. If a consensus could not be reached, a third reviewer was consulted for arbitration. After removing duplicates and screening titles and abstracts, 109 articles were retained for full-text review. To ensure comprehensive coverage, the reference lists of relevant articles were also manually searched in addition to the database searches. Ultimately, seven studies met the predefined inclusion criteria. The study selection flow is detailed in Fig. 1, which was created following the Preferred Reporting Items for Systematic Reviews and Meta-Analyses (PRISMA) statement [22].

### Quality assessment

**Fig. 2:**
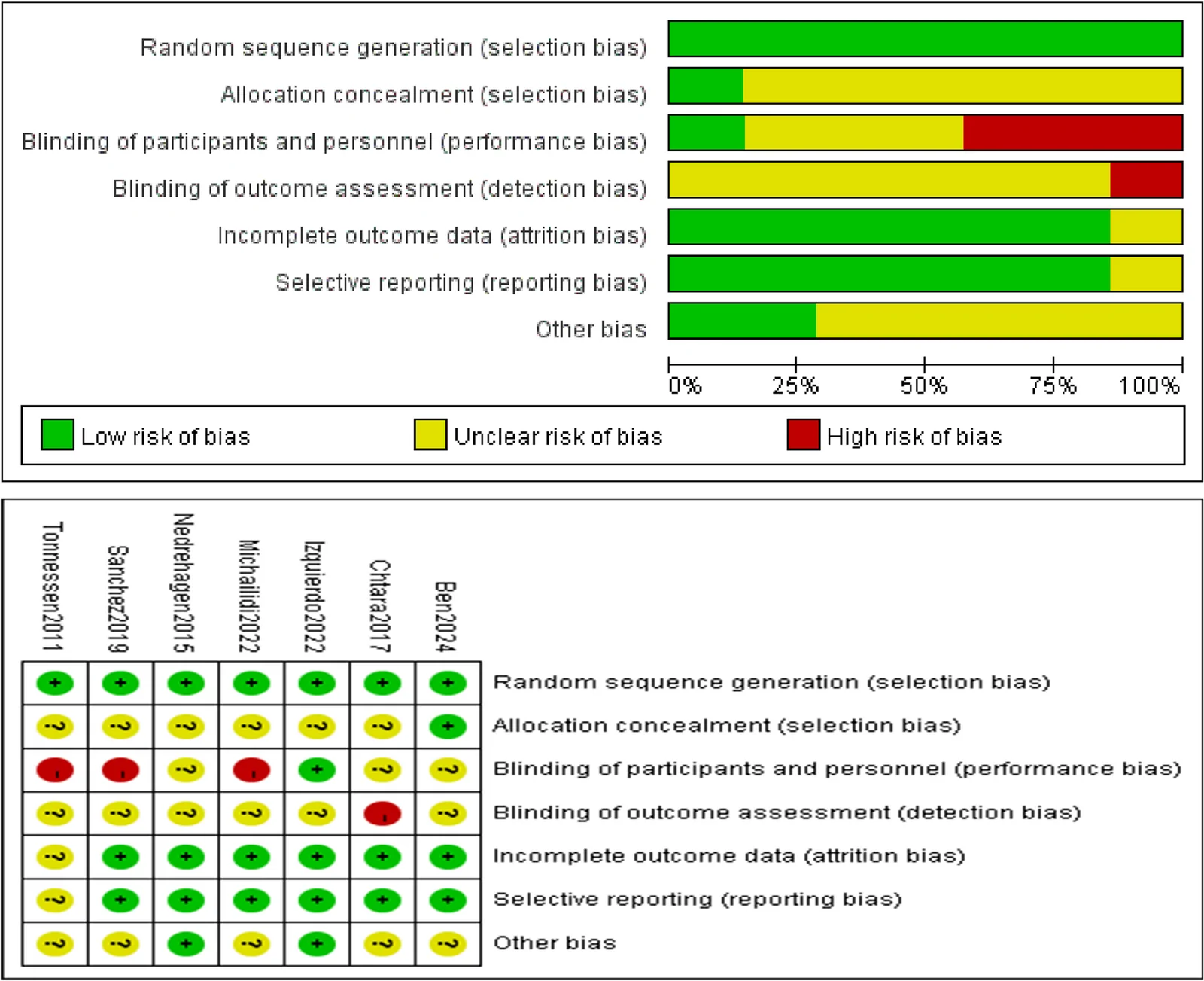
Cochrane risk of bias assessment

The methodological quality and risk of bias of the included studies were assessed using the Cochrane Risk of Bias tool [23]. The evaluation was performed with Review Manager 5.4 software, focusing on the following domains: random sequence generation, allocation concealment, blinding of participants and personnel, blinding of outcome assessment, incomplete outcome data, selective reporting, and other potential sources of bias. Each domain was judged as being at “low risk”, “high risk”, or “unclear risk” of bias. Two reviewers independently conducted the quality assessment. Any disagreements or uncertainties were resolved through consultation with a third expert to ensure the accuracy of the judgments. The results of the risk of bias assessment are presented in Fig. 2.

### Statistical analysis

Data synthesis and meta-analysis were performed using Review Manager 5.4 (RevMan). For continuous outcome measures, which were assessed using varying methods and units across studies, the Standardized Mean Difference (SMD) with 95% confidence intervals (CI) was selected as the summary effect measure. Data extracted for analysis included the mean, standard deviation, and sample size from each group. Heterogeneity among the included studies was quantified using the *I*^*2*^ statistic. A fixed-effect model was applied when *I*^*2*^ was ≤ 50%, indicating low heterogeneity; otherwise, a random-effects model was used. The pooled effect estimates are presented in forest plots, while potential publication bias was assessed using funnel plots. To explore potential sources of heterogeneity, subgroup analyses were conducted based on athlete level, age, intervention duration, and outcome measures.

## Research

### Inclusion of basic characteristics of the literature

A total of seven studies were included [24–30], all of which were from European countries. Six of these studies exclusively involved male subjects [24–26, 28−30], with one study [27] involving mixed-gender participants. Additionally four studies featured elite athletes [24, 26, 29, 30], three studies involved non-elite athletes [25, 27, 28]. The total sample size was 202 participants, with 23 dropouts due to injury or absence, resulting in an effective sample size of 179. The mean sample size per study was 25.6 ± 4.1 participants, with a mean age of 17.3 ± 3.1 years. Additionally, six studies [24–26, 28−30] explicitly excluded goalkeepers. One additional study [27] included subjects from multiple positions. Subject characteristics across the included studies are summarized in Table 2.

**Table 2:** Characteristics of subjects in included studies

Author(Year)	Country	Gender	Sample size(persons)	Age (years)	Level	Cycle (weeks)	Frequency/week
Chtara 2017 [14]	Tunisia	Male	22	13.6±0.3	Elite athletes	6	2 times
Izquierdo 2022 [15]	Spain	Male	27	17.7±0.7	Non-elite athletes	4	3 times
MichailidI 2022 [16]	Greece	Male	29	15.9±0.9	Elite athlete	4	2 times
Nedrehagen 2015 [17]	Norway	Male/Female	22	21.0±2.9	Non-elite athletes	8	1 session
Sanchez 2019 [18]	Spain	Male	29	14.65±0.14	Non-elite athlete	8	2 times
Tonnessen 2011 [19]	Norway	Male	20	16.4±0.9	Elite athlete	10	1 session
Ben 2024 [20]	Tunisia	Male	30	21.8±2.6	Elite athlete	8	3 times

### Repeated sprint training intervention protocols

Among seven studies [24–30] cited, the average intervention period was 6.2 ± 1.9 weeks, with an average total of 11 ± 2.5 sessions and an average weekly frequency of 1.9 ± 0.5 sessions. Most exercise formats employed fixed-distance sprint runs, though RST protocols varied across studies in terms of training intensity, volume, and rest duration. Regarding intensity, most studies utilized maximal-intensity sprints; one study [29] employed 95%–100% of maximal intensity. Training volume comprised sets, repetitions, and sprint distance: sets ranged from 2 to 5, repetitions from 4 to 10, and sprint distance from 20 to 40 m. Inter-set duration was approximately 15 s to 2 min, with inter-set rest periods of 1 to 10 min. Characteristics of the intervention programs in the included studies are summarized in Table 3.

**Table 3:** Characteristics of intervention programs in included studies

Author (Year)	Load Intensity	Training Volume (Sets × Repetitions)	Rest Period	Outcome Measures
Chtara 2017 [14]	100%	(3–5) × (5–6) sets of 40 m	20 s rest within sets, 4 min rest between sets	①②③
Izquierdo 2022 [15]	100%	2 × 8 reps (10–30 m)	10–15 s inter-set, 8 min inter-set	①②③
MichailidI 2022 [16]	100%	(2–4) × 6 sets 40 m	20 s inter-set, 4 min inter-set	①②③
Nedrehagen 2015 [17]	100%	(3–4) × (4–6) 30 m	30 s inter-set, 5 min inter-set	②
Sanchez 2019 [18]	100%	3 × 10 reps 18 m	25 s inter-set, 4 min inter-set	①②
Tonnessen 2011 [19]	95 ~ 100%	(2–5) × (4–5 sets) 40 m	1.5–2 min inter-set, 10 min inter-set	①②③
Ben 2024 [20]	100%	2 × (5–10 reps) 20 m	15 s inter-set, 1 min inter-set	①②③

Outcome measures: ① RSA (best), ② RSA (mean), ③ Short-distance sprint”

### Control group intervention protocol

In this meta-analysis, the control groups across seven studies [24–30] all employed conventional training programs. These primarily comprised low-to-moderate intensity technical drills such as ball control, passing, dribbling, and feints. They also incorporated tactical training, some agility and coordination exercises, and simulated match practice. Technical and tactical sessions were conducted at a relatively low intensity, with a primary focus on developing players’ technical proficiency and tactical coordination. Some training loads reached moderate to high intensity; however, the duration of rest periods and training volume were not explicitly detailed in the literature. The comparison of training programs between experimental and control groups across seven studies [24–30] is shown in Table 4.

**Table 4:** Comparison of training protocols for experimental and control groups

Author (Year)	Experimental Group Training Protocol	Control Group Training Protocol	Cycle (weeks)	Frequency (Week)
Chtara 2017 [14]	(3–5) × (5–6) sets of 40 m	Technical drills + tactical work + games + simulated matches	6	2 sessions
Izquierdo 2022 [15]	2 × 8 sets (10–30 m)	Technical + Tactical + Small-sided Games + Recovery Runs	4	3 sessions
MichailidI 2022 [16]	(2–4) × 6 sets of 40 m	Technical + Tactical + Small-Sided Games + Fitness	4	2 sessions
Nedrehagen 2015 [17]	(3–4) × (4–6) 30 m	Low-to-medium intensity technical + tactical training	8	1 session
Sanchez 2019 [18]	3 × 10 reps 18 m	Technical + Tactical + Small-sided Games	8	2 sessions
Tonnessen 2011 [19]	(2–5) × (4–5 reps) 40 m	Technical + Tactical + Small-Sided Games + Core Strength	10	1 session
Ben 2024 [20]	(3–5) × (5–6) sets of 40 m	Technique + Tactics + Fitness + Match Simulation	8	3 sessions

### Meta-analysis

#### Repeated sprint maximum speed

**Fig. 3:**
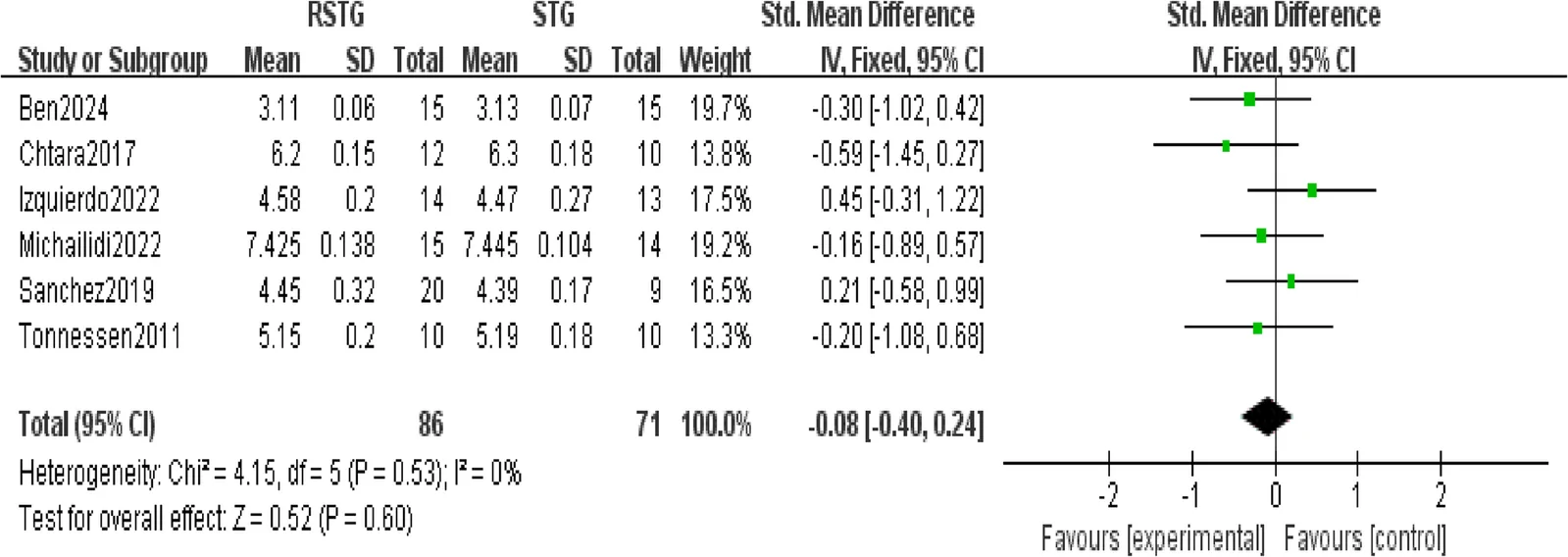
Meta-analysis forest plot of RST effects on RSA_best_

In the included studies, a total of six studies [24–26, 28−30] reported the effect of RST on RSA_best_. Sanchez et al. [28] divided RST into low oxygen group and high oxygen group, while the rest of the studies were randomized. Therefore, the two experimental groups of Sanchez et al. [28] were merged to calculate the RSA_best_ of random RST as 4.45 ± 0.32 s [28]. There is no heterogeneity among the studies (*I²* = 0%, *p* = 0.53), therefore a fixed effects model was used for merging, as shown in Fig. 3. The meta-analysis results showed that the effect of RST on RSA_best_ was not significant (*SMD* = -0.08, 95% *CI*: -0.40 to 0.24, *p* = 0.600). From the forest plot, the confidence interval includes zero and is slightly skewed to the left, indicating that the improvement effects of the two groups on RSA_best_ tend to be similar, although RST may have a slight advantage.

Adjusted analyses for different moderating variables were conducted across six [24–26, 28−30]. As shown in Table 5, only minor heterogeneity was observed in subgroups with intervention duration ≤ 6 weeks, RSA outcome measure of 40 m, and athlete mean age ≤ 16 years; no heterogeneity existed among other subgroups (*I²* = 0%). Therefore, based on the results of subgroup comparisons, most differences between subgroups were not statistically significant. These two training methods may not significantly differ in their effects on athletes’ RSA_best_.

**Table 5:** Subgroup analysis of RST effects on RSA_best_

Subgroup	Type	Number	SMD	95%CI	*P*	I²(%)
Level of physical activity	elite Non-elite	4 2	-0.30 0.33	-0.69–0.10 -0.22–0.88	0.140 0.240	0 0
Intervention period	≤ 6 weeks > 6 weeks	3 3	-0.06 -0.10	-0.51–0.39 -0.56 – -0.35	0.780 0.650	38 0
Average age	≤ 16 years > 16 years	3 3	-0.16 -0.01	-0.61–0.30 -0.46–0.44	0.500 0.950	0 9
RSA distance	40 M 20、30 M	3 3	0.05 -0.22	-0.55–0.65 -0.67–0.22	0.870 0.330	39 0

#### Average speed in repeated sprints

**Fig. 4:**
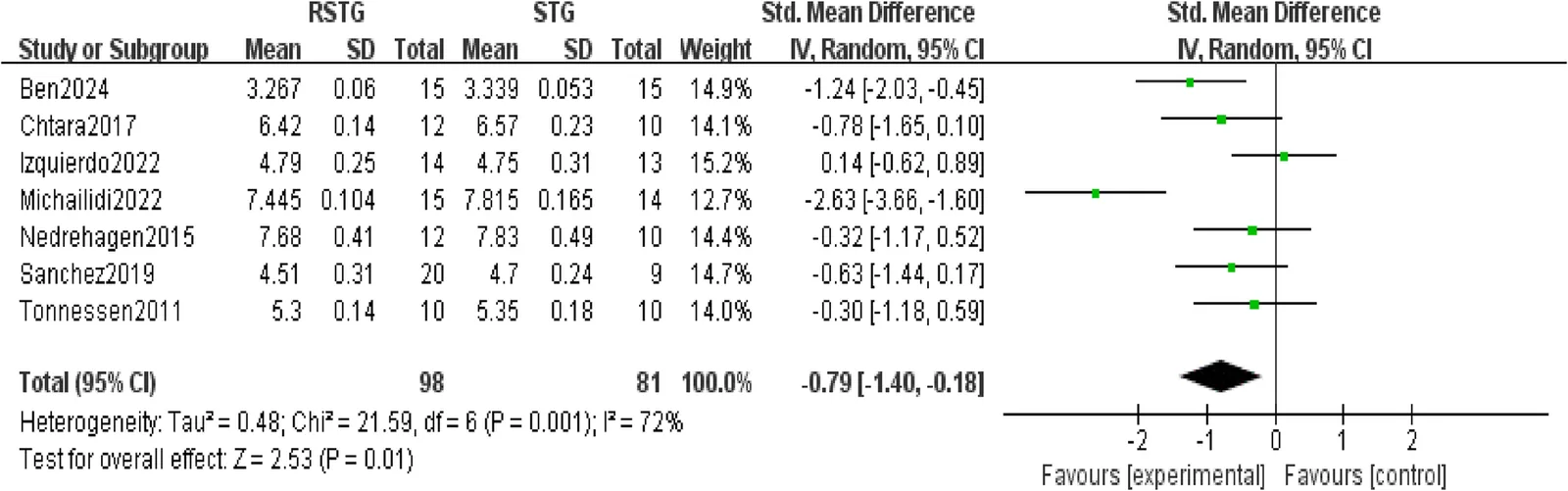
Meta-analysis forest plot of RST effects on RSA_mean_

Seven studies in total [24–30] reported on the effect of RST on RSA_mean_. Sanchez et al. [28] divided RST into hypoxic and hyperoxic groups, whilst the remaining studies employed randomized experimental groups. Consequently, the two experimental groups from Sanchez et al. [28] were combined. Calculations yielded the following mean outcome measures for the randomized experimental groups: RSA_mean_ 4.51 ± 0.31 s [28]. High heterogeneity existed between studies (*I²* = 72%, *p* = 0.001), necessitating a random-effects model for effect size pooling. The forest plot is presented in Fig. 4, yielding an SMD of -0.79 with a 95% confidence interval of -1.40 to -0.18. The p-value of 0.01 indicates no statistically significant difference.

To further investigate the sources of heterogeneity, subgroup analyses were conducted based on different moderating variables. As shown in Table 6, subgroup analyses for RSA_mean_ revealed substantial heterogeneity in the 40 m distance, under-16 age group, elite athletes, and intervention period ≤ 6 weeks subgroups. In subgroups such as elite athletes and intervention periods > 6 weeks, RST demonstrated markedly superior enhancement of RSA_mean_ compared to conventional training. Within subgroups exhibiting greater heterogeneity—specifically 40-metre distance, under 16 years of age, and elite athletes—RST occasionally outperformed conventional training. Subgroup analysis further revealed that the subgroup with intervention periods exceeding 6 weeks exhibited optimal performance in terms of both effect size significance and low heterogeneity (*I²* = 9%). This indicates that within this subgroup, RST delivers stable and significant improvements in RSA_mean_ with minimal variability. Significant effect ( *p* = 0.004) with low heterogeneity (*I²* = 9%), indicating stable and pronounced RST enhancement of RSA_mean_ in this subgroup. In contrast, while some results were significant in subgroups such as 40 m sprint distance, under-16 s, and elite athletes, *I²* values reached 86%, 80%, and 76% respectively. This indicates heterogeneity primarily stemmed from differences in test distance, developmental stage, and training background. Furthermore, substantial variations in key parameters such as total sprint repetitions (4–10), inter-set rest intervals (15–120 s ), and training frequency (1–3 sessions/week) across these studies likely contributed significantly to the overall effect size variability.

**Table 6:** Subgroup analysis of RST effects on RSA_mean_

Subgroup	Type	Number	SMD	95%CI	*P*	I(%)
Level of physical activity	elite Non-elite	4 3	-1.21 -0.25	-2.21 – -0.31 -0.71–0.21	0.009 0.280	76 0
Intervention period	≤ 6 weeks >6 weeks	3 4	-1.06 -0.65	-2.58–0.47 -1.08 – -0.21	0.180 0.004	89 9
Average age	≤16 years >16 years	3 4	-1.31 -0.43	-2.48 – -0.14 -1.02–0.16	0.030 0.160	80 53
RSA distance	40 M 20、30 M	3 4	-1.06 -0.62	-2.48–0.36 -1.20–0.03	0.140 0.040	86 53

#### Short-distance sprinting ability

**Fig. 5:**
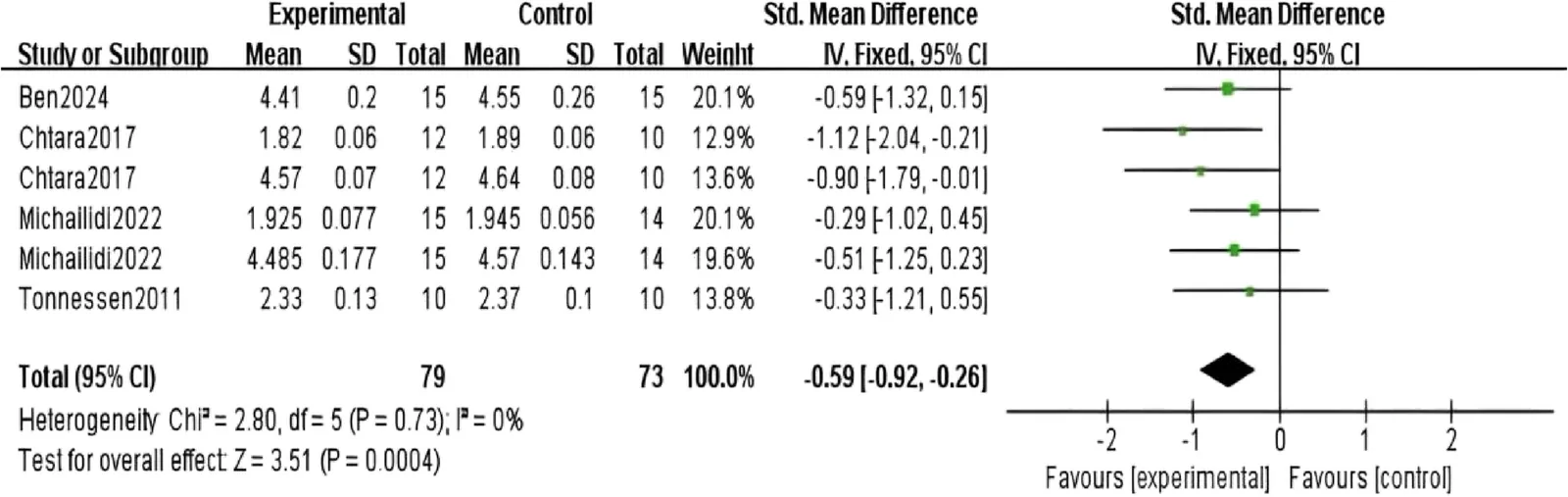
Meta-analysis forest plot of RST effects on short-distance sprinting ability

Among the included studies, four investigations [26, 27, 29, 30] examined the impact of RST on short-distance sprinting ability, encompassing 10 m, 20 m, and 30 m sprints. Heterogeneity tests revealed no significant variation between studies (*I*^*2*^ = 0%), permitting analysis using a fixed-effects model. The meta-analysis yielded a *SMD* of -0.59, 95% confidence interval of -0.92 to -0.26, *p* < 0.001, as illustrated in Fig. 5. This indicates that RST exerts a significantly different effect on short-distance sprinting capacity compared to conventional training. Furthermore, the absence of heterogeneity among studies lends greater reliability to the pooled results. In practical training contexts, RST may be considered to effectively enhance soccer players’ short-distance sprinting capabilities.

### Publication bias and sensitivity analysis

Conducting publication bias and sensitivity analyses on the included studies tests the completeness of meta-analysis inclusion while evaluating the stability of results. Publication bias was assessed using funnel plots generated via Review Manager 5.4 software. Results indicated that all funnel plots exhibited good symmetry, suggesting a low likelihood of publication bias among the included studies. Sensitivity analysis was performed by sequentially excluding studies or literature and including other excluded literature. It was found that excluding certain studies in the research on the effect of RST on anaerobic sprint capacity led to changes in the outcome. Therefore, the reliability of the conclusion regarding the effect of RST on anaerobic sprint capacity needs to be considered.

## Discussion

### Effects of repeated sprint training on maximum speed in repeated sprints among soccer players

Meta-analysis results indicate that RST does not significantly improve RSA_best_, showing no significant difference compared to conventional training (*SMD* = -0.08, *p* = 0.600), with some individual studies demonstrating negative effects. This finding aligns with certain previous research outcomes. Izquierdo et al. [25] noted that RST significantly enhances RSA_mean_ in soccer players, but its effect on RSA_best_ [25]. Ben et al. [30]. further supported this observation, demonstrating that RST significantly improved both RSA_mean_ and RSA_best_, though the magnitude of improvement was greater for RSA_mean_ and the effect was more pronounced [30]. From an energy metabolism perspective, the core reason lies in RSA_best_ being dependent on the instantaneous power output of a single sprint, primarily relying on the instantaneous power output of the phosphocreatine (ATP-PCr) system during the first 5–6 s of a 30–40 m sprint. While the “multiple incomplete recovery” pattern of repeated sprint training can also enhance certain ATP-PCr reserves, the direct stimulation of ATP-PCr reserves remains insufficient [31, 32]. Consequently, the fatigue accumulation advantage of RST fails to translate into improvements in single-repetition maximum speed [17]. As the number of sprints increases, PCr recovery becomes constrained, power output diminishes, and the contribution of the glycolytic system in this process consequently rises [33]. However, RST exerts a relatively weak direct effect on enhancing glycolytic energy supply during RSA_best_, and its training impact requires the accumulation of fatigue through multiple sprints to gradually manifest. Consequently, the training effect of RST is primarily manifested through RSA_mean_, which enhances the storage and resynthesis efficiency of energy substrates while strengthening the buffering capacity of fatigue-related substances. This reflects the glycolytic system’s fatigue resistance and recovery efficiency during rest intervals (e.g., lactate clearance, phosphocreatine resynthesis), constituting the principal improvement direction of RST. Buchheit et al. [34] investigated the effects of RST and explosive strength training on the athletic performance of adolescent soccer players. Results revealed that explosive training yielded comparable effects to repeated sprint training in enhancing athletes’ maximum speed [34]. Suarez-Arrones et al. [35] compared the effects of RST versus combined RST and explosive training on athletic performance. Findings indicated that the combined RST and explosive approach produced more pronounced improvements in athletes’ RSA_best_ and relative explosive power [35]. Therefore, future training should specifically enhance ATP-CP reserves (e.g.,ultra-short interval sprints) or integrate compound training (e.g., RST + explosive strength) to improve RSA_best_.

### Effects of repeated sprint training on average speed during repeated sprints in soccer players

RSA is consistent with soccer match patterns and the energy metabolism characteristics during gameplay, accurately reflecting the anaerobic sprinting capacity of players across different levels and positions. Consequently, RSA is commonly employed in soccer to assess athletes’ anaerobic capabilities, recognised as a crucial attribute in team ball sports [36, 37]. Meta-analysis shows that RST has a more significant improvement effect on RSA_mean_ compared to conventional training (*SMD* = -0.79, *p* = 0.010), with lower heterogeneity observed in longer-duration interventions (> 6 weeks) (*I²* = 9%, *p* = 0.004), effectively enhancing soccer Players’ RSA_mean_. The study by Tonnensen et al. [29] unequivocally demonstrated that following a 10-week RST programme, soccer Players’ RSA_mean_ shows significant improvement( *p* ≤ 0.010) [29]. The study by Nedrehagen et al. [27] found RST reduced the time to reach mean RSA by 1.5% [27]. Furthermore, multiple studies indicate RST significantly enhances mean RSA, whereas conventional training yields relatively minor or non-significant improvements [24–30]. During RST at ≥ 90% maximum sprint speed, the ATP-PCr system bears the primary power output, with relatively limited glycolytic mobilisation. Initial lactate production is not high; lactate accumulation arises when clearance rates temporarily fall below production rates [38]. Long-term RST progressively enhances the efficiency of lactate recycling and clearance by upregulating monocarboxylate transporters and mitochondrial oxidase activity. This delays power decay during subsequent sprints [14, 39], enabling muscles to sustain higher glycolytic rates and thereby improve the ability to maintain speed during high-intensity exercise [40, 41]. Furthermore, all seven included studies employed incomplete recovery between training sets in their experimental groups, effectively stimulating CP resynthesis rates and prolonging high-intensity exercise duration [24–30]. Research indicates that PCr resynthesis within human muscle follows a double-exponential kinetics. Following a single 6-second sprint, only approximately 60% recovers within 90 s, with full recovery requiring 3–5 min. Moreover, the recovery rate positively correlates with local O₂ availability [42]. Consequently, RST subjects PCr to a “partial recovery-partial depletion” cycle, compelling type II muscle fibers to repeatedly generate high-power outputs at low PCr thresholds. This constitutes the core metabolic lever for enhancing average power during repeated sprints. The enhancement of RSAmean represents a synergistic adaptation of metabolic, neural, and energy systems. Long-duration (> 6 weeks), high-load (≥ 90% intensity) RST can strengthen soccer player repeated sprint capacity. Repeated sprint training may prove a more effective approach. However, individual variations and specific training conditions must be considered; training plans should be tailored and optimised according to each athlete’s circumstances.

### Effects of repeated sprint training on short-distance sprinting ability in soccer players

RST has garnered significant attention as an effective method for specifically enhancing sustained explosive power [4, 5, 14]. Through multiple short-distance, high-intensity sprints, RST primarily enhances repeated-sprint ability and short-distance sprint performance—qualities that allow soccer players to accelerate, decelerate, and change pace more effectively during match play, thereby conferring a competitive advantage [43, 44]. For example, by allowing a player to win a decisive sprint to reach a through ball, track back more effectively to intercept an attacking opponent, or execute multiple high-intensity runs in quick succession during the final minutes of a half when fatigue typically impairs performance [45–47]. RSA, as a crucial physical attribute for soccer players, has always been highly regarded [14, 48]. Meta-analysis findings indicate that RST significantly enhances soccer players’ short-distance sprinting capacity (*SMD* = -0.59, *95% CI*: -0.92 to -0.26, *p* < 0.001). Such improvements are primarily governed by neural mechanisms. Early-phase rate of force development (RFD) predominantly reflects neural drive, with power-focused modalities improving motor unit recruitment thresholds and firing frequency, particularly in high-threshold motor units [49–51]. Furthermore, the speed of motor unit recruitment has been identified as a primary limiting factor for maximal RFD, with faster recruitment intervals directly correlating with greater explosive force output [52, 53]. In this context, RST—characterized by maximal-intensity, short-duration sprint efforts—demonstrates the capacity to activate these neuromuscular adaptations, lowering the recruitment threshold for high-threshold type II fibers and increasing force development rate, thereby optimising acceleration and initial speed [14].

In soccer matches, athletes frequently execute short-distance sprints, with sprinting ability often determining the final outcome of a match [54]. Short-duration, short-distance sprints primarily rely on anaerobic metabolism for energy supply and the CP’s capacity to resynthesis ATP. Training should employ methods involving short time, high intensity, full recovery, and repetition [55, 56]. During RST, sustained high-speed levels are maintained repeatedly to deliver continuous stimulation to muscular strength, power, and coordination [57–59]. While these neuromuscular adaptations can occur in both trained and untrained individuals, the magnitude of improvement is generally more pronounced in those with a lower initial training base (e.g., youth or non-elite players), whereas elite athletes may experience more modest gains primarily in repeated-sprint economy rather than in maximal strength or power [60]. This enhances motor unit recruitment frequency and lowers recruitment thresholds, reducing the curvature of the force-velocity curve. It also improves energy storage and elastic potential release, thereby elevating overall athletic performance [14, 61]. RST stimulates increased mitochondrial enzyme activity within muscles, thereby enhancing phosphocreatine resynthesis and muscle glycogen storage while reducing glycogen utilization and lactate accumulation [14]. During RST training, athletes must continuously accelerate, decelerate, and change direction, more closely simulating the metabolic demands of soccer matches. Consequently, RST may hold significant promise for enhancing match-related performance in soccer players [62]. Although RST markedly improves soccer players’ short-distance sprint performance, its effects are influenced by multiple factors, including: (1) initial fitness level (e.g., elite vs. non-elite) [61]; (2) RST protocol variables such as sprint distance, rest ratio, and total volume (see Table 3) [24–30]; (3) intervention duration (≤ 6 weeks vs. >6 weeks, as shown in our subgroup analyses) [24–30]. Future research should incorporate multi-directional change-of-direction sprints to more comprehensively replicate the practical demands of competitive soccer.

## Conclusions

1. For strength and conditioning coaches working with youth soccer players, integrating RST into the weekly training schedule is recommended. Based on our subgroup analyses, an optimal protocol to enhance RSAmean and short‑distance sprint performance consists of: Sprint distance: 40 m; Training frequency: 2 sessions per week; Intervention duration: ≥ 6 weeks (longer protocols produced more stable improvements; *I*² = 9%, *p* = 0.004); Session volume: 4–6 repetitions × 2–4 sets (total 24–30 sprints); Work‑to‑rest ratio: approximately 1:5 to 1:10 (e.g., 20 s rest between reps, 4 min rest between sets).
2. RST significantly enhanced RSAmean by improving glycolytic energy supply and lactate metabolism in soccer players, and RSAbest may be further optimized by combining RST with other explosive power training. Subsequent studies should refine classification metrics.
3. Existing RST protocols showed no significant improvement in repeated sprint maximum speed (RSAbest) (pooled *SMD* = -0.08, *p* = 0.60). Where training objectives involve maximum speed, additional short sprints should be designed with priority given to the phosphate system (≤ 5 s) and full recovery, or combined explosive training should be incorporated. This provides scientific rationale for designing anaerobic training for soccer players.
4. Due to limitations in language, gender, position, and insufficient dosage reporting, these conclusions primarily apply to male elite youth players. Caution is advised when extrapolating findings to female or adult professional populations. Future research should account for the influence of factors such as gender, position, and environment to achieve truly precise training prescriptions.

## Supplementary Information

Supplementary Material 1.

## Data Availability

The data extracted for this systematic review and meta-analysis, as well as the analytic code used, are available from the corresponding author upon reasonable request.
